# An Observational Study of Evidence-Based Therapies in Older Patients with Heart Failure with Reduced Ejection Fraction: Insights from a Dedicated Heart Failure Clinic

**DOI:** 10.3390/jcm13237171

**Published:** 2024-11-26

**Authors:** Catarina Silva Araújo, Irene Marco, María Alejandra Restrepo-Córdoba, Isidre Vila Costa, Julián Pérez-Villacastín, Josebe Goirigolzarri-Artaza

**Affiliations:** 1 Internal Medicine, Braga Hospital, 4710-243 Braga, Portugal; catarinaaraujo.mi@gmail.com; 2 Cardiovascular Institute, Instituto de Investigación Sanitaria, Hospital Clínico San Carlos (IdISSC), C/Prof Martín Lagos S/N, Moncloa-Aravaca, 28040 Madrid, Spain; irene.marco@salud.madrid.org (I.M.);

**Keywords:** heart failure, guideline-directed medical therapies, geriatric cardiology

## Abstract

**Background/Objectives:** Despite significant advances in the management of heart failure with reduced ejection fraction (HFrEF), data concerning older patients remain limited. The purpose of this study was to evaluate the implementation of guideline-directed medical therapy (GDMT) in older patients with HFrEF along with cardiac events and variation in clinical and echocardiographic parameters during follow-up in a heart failure (HF) clinic. **Methods:** We conducted a retrospective observational analysis of patients with HFrEF aged ≥ 80 years who attended an HF clinic between March 2022 and February 2023. The primary outcome was a composite of the first episode of worsening HF or cardiovascular death. All-cause death was also recorded. **Results:** We included 110 patients (30.9% females; mean age 82.9 years). After a median follow-up of 25.5 months, left ventricular ejection fraction (LVEF) improved (mean difference 12.5% (*p* < 0.001)). New York Heart Association class improved in 37% of patients, and N-terminal pro-B-type natriuretic peptide levels decreased (3091 (158–53354) to 1802 (145–19509), *p* < 0.001). The primary outcome occurred in 34 patients (30.9%). Patients without the primary outcome were more likely to receive sodium-glucose co-transporter-2 inhibitors (SGLT2i) (23.5% versus 67.1%, *p* < 0.001) and angiotensin receptor-neprilysin inhibitors, angiotensin-converting enzyme inhibitors, or angiotensin-receptor blockers (67.6% versus 84.2%, *p* < 0.05). These patients also received a greater number of GDMT medications (2 (0–4) versus 3 (1–4), *p* < 0.01) and demonstrated a higher LVEF at the last visit (41.2 ± 10.2% versus 47.1 ± 9.4%, *p* < 0.05). Survival analysis demonstrated a significant association between LVEF recovery (hazard ratio (HR) 0.35, *p* < 0.01), treatment with two or more GDMT medications (HR 0.29, *p* < 0.01), vasodilator use (HR 0.36, *p* < 0.01), and SGLT2i prescription (HR 0.17, *p* < 0.001) and a reduced risk of the primary endpoint. **Conclusions:** The optimization of HF treatment is achievable in older patients and may be associated with a reduction in cardiac events.

## 1. Introduction

Heart failure (HF) is associated with significant morbidity and mortality, and its prevalence exponentially increases with age, reaching 10% in those over 80 years old. This condition has emerged as a significant challenge in the elderly population, representing the leading cause of hospitalization in this age group [1,2,3]. More than half of those hospitalized for HF are over 75 years old. The prognosis also worsens with age, with 1- and 5-year mortality rates of 19.5% and 54.4% for 80 year olds [4]. While preserved ejection fraction is more prevalent in older individuals with HF, a considerable proportion presents with reduced ejection fraction (HFrEF) [5].

Advances in HFrEF diagnosis and management have shown great reductions in cardiovascular events, hospitalizations, and mortality in clinical trials over the last few decades [6]. In view of these results, international guidelines have endorsed the so-called “4 pillars” for HFrEF treatment: angiotensin receptor-neprilysin inhibitor—ARNI, or angiotensin-converting enzyme inhibitor—ACE-I); beta-blockers; mineralocorticoid receptor antagonist (MRA) and sodium-glucose co-transporter 2 inhibitors (SGLT2i) (dapagliflozin/empagliflozin) [1,7,8]. However, the use of guideline-directed medical therapy (GDMT) can be challenging in this population. There are limited data about HFrEF treatment in older patients as they are underrepresented in major randomized clinical trials [9,10,11]. In addition, clinical trials aimed at this specific population are marginal [12]. Also, clinicians can be overcautious in the presence of multiple comorbidities and polypharmacy. HF clinics may offer a means of enhancing the quality of care for this population, facilitating close follow-up and meticulous titration of GDMT [13].

This study aimed to evaluate the degree of implementation of HF therapies in an older population with HFrEF, assessing clinical and echocardiographic changes and documenting the occurrence of cardiac events during follow-up within an HF clinic.

## 2. Materials and Methods

### 2.1. Patient Selection and Study Design

This study was an observational, single-center retrospective analysis of consecutive elderly patients diagnosed with HFrEF who visited a tertiary hospital heart failure clinic led by cardiologists during one year (from March 2022 to February 2023). The selection of this timeframe ensures a standardized management of HFrEF following the ESC practice guidelines of HF issued in late 2021 [1]. Patients that were 80 years old or older at the time of first visit and were at least 75 years old at the time of HF diagnosis were included. Exclusion criteria included severe primary valvular heart disease requiring intervention, a life expectancy of less than 6 months, and a follow-up period of less than 6 months in the HF clinic. This study was conducted in compliance with the Declaration of Helsinki and reviewed by the Institutional Review Board Ethical Committee of our center (24/720-E).

### 2.2. Data Collection and Follow-Up

Data from the first and last clinical evaluation were retrospectively collected. Demographic, clinical, laboratory, echocardiographic, and electrocardiographic data were recorded. Echocardiographic parameters registered included left ventricular ejection fraction (LVEF), left ventricular end-diastolic diameter (LVEDD), mitral valve regurgitation, and the presence of right ventricular dysfunction. The New York Heart Association (NYHA) classification was used to assess functional class. Glomerular filtration rate (GFR) was estimated with Chronic Kidney Disease Epidemiology Collaboration (CKD-EPI) equation. LVEF at diagnosis and the suspected etiology of HF were also registered.

A ≥10-point increase from diagnosis with a final LVEF of >40% at the last visit was considered improved LVEF, and LVEF recovery was defined as an increase in LVEF of at least 10% to a value ≥50%. Primary outcome was defined as the composite of the first episode of worsening HF (HF hospital admission or need for intravenous diuretics in the outpatient clinic) or cardiovascular death. All-cause death was also addressed. HF treatments at the time of the first episode of worsening HF and one month after its occurrence were also registered.

All required information was extracted from the electronic records system and compiled into an anonymized Excel sheet using a standardized extraction procedure, which included validation rules. Medical charts were revised to ensure their reliability and accuracy before data extraction. When multiple sources were available, cross-checking was performed to identify any discrepancies. A single individual conducted data extraction, while a different expert reviewed the extracted data to ensure accuracy prior to analysis.

### 2.3. Statistical Analysis

The collected data were analyzed using the software IBM SPSS^®^ version 23.0 and GraphPad Prism 10^®^. Pie charts were designed with Microsoft Excel^®^. The Kolmogorov–Smirnov test was used to test the existence of a normal distribution in continuous quantitative variables. Continuous variables with a normal distribution were presented as mean and standard deviation. The median, interquartile range, and minimum and maximum values were used for those with a non-normal distribution. Categorical variables were presented as absolute numbers and percentages. Between-group analyses of categorical variables were performed with the chi-square, Fisher’s exact, and McNemar tests. The Kruskal–Wallis, Man–Whitney U, Wilcoxon, Spearman, and paired samples *t*-tests were used for continuous variables. Analysis of binary outcomes such as LVEF recovery was performed with logistic regression, using a backward stepwise approach for multivariate analysis with *p* = 0.05 for inclusion and *p* = 0.10 for exclusion. Time-to-first-event curves were obtained using the Kaplan–Meier method and compared with log-rank test. Hazard ratios (HR) were obtained using univariate Cox regression analysis, including predefined variables (GDMT, LVEF recovery). A 95% confidence interval was considered, with statistical significance at *p* < 0.05.

## 3. Results

### 3.1. Sample Description

This study included 110 patients (30.9% females; 80 ± 6 years at HF diagnosis and 82 ± 4 years at first evaluation). Baseline characteristics are described in Table 1. Diabetes was present in 41.8% of patients and chronic kidney disease in 50.9%. Mean LVEF at diagnosis was 32.4%, with the most common cause of HF being non-ischemic dilated cardiomyopathy (37.3%), followed by ischemic cardiomyopathy. Two-thirds of the patients included in this study were referred to the HF clinic after a hospital admission for HF.

### 3.2. Follow-Up

The median follow-up time was 25.5 months. Differences between first and last visits are described in Table 2.

**Figure 1:**
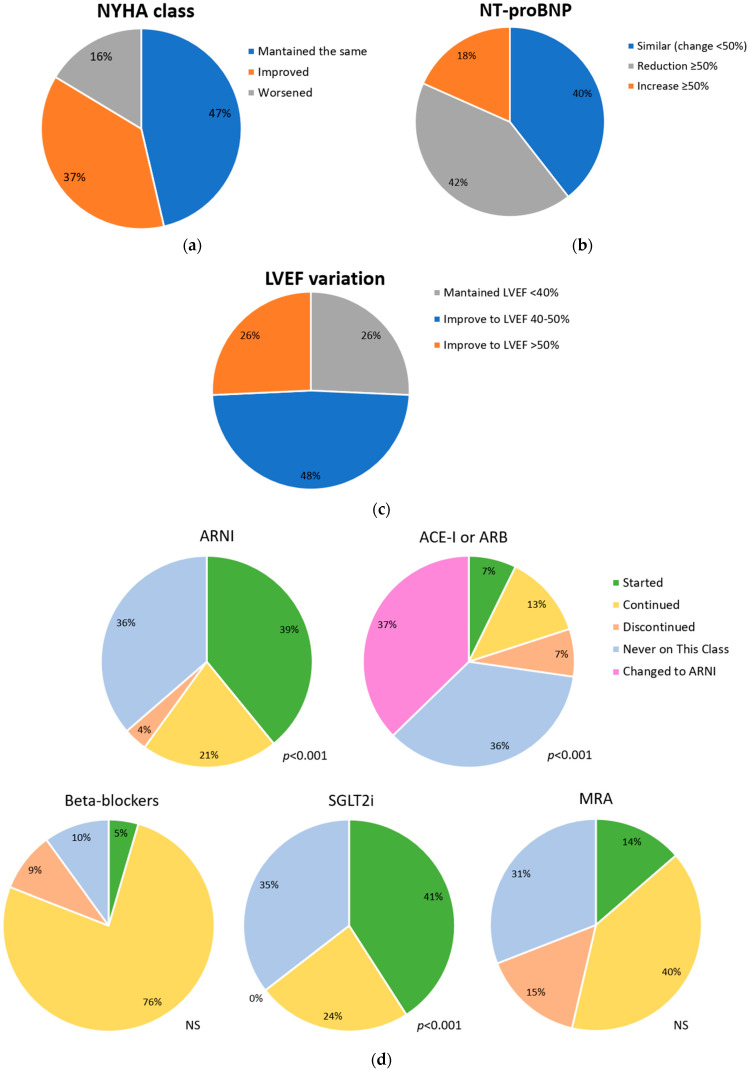
Variation in NYHA class (**a**), NT-proBNP levels (**b**), LVEF (**c**) and medical therapies (**d**) from first to last visit at the Heart Failure Clinic. ACE-I—angiotensin-converting enzyme inhibitor; ARB—angiotensin-receptor blocker; ARNI—angiotensin receptor-neprilysin inhibitor; LVEF—left ventricle ejection fraction; MRA—mineralocorticoid receptor antagonist; NYHA—New York Heart Association; NS—non-significant; SGLT2i—sodium-glucose co-transporter 2 inhibitor.

At the initial visit, over half of the patients were in the NYHA class II (60%), while 22.7% were in the NYHA class III and 17.3% in the NYHA class I. A significant improvement in NYHA functional class was observed during follow-up (Figure 1). Specifically, 37% of patients improved to a better functional class, and 27.3% were in the NYHA class I (*p* < 0.05) at the last visit. Atrial fibrillation and left ventricular bundle branch were described at first visit in 40% and 26% of patients, with no significant differences during follow-up. NT-proBNP levels significantly decreased during follow-up (median 3091 vs. 1802, *p* < 0.001), and a significant reduction was also observed in estimated GFR (54.5 ± 18.0 vs. 47.2 ± 14.8, *p* < 0.001).

LVEF significantly improved from first to last visit, with an absolute difference of 12.5% (*p* < 0.001). Improved LVEF was observed in 53% of patients, and 27% showed LVEF recovery. Grade III or IV mitral regurgitation was present in 19.1% of patients at first visit, with a significant reduction to 5.7% at last visit (*p* < 0.001). No significant changes were observed in right ventricular ejection fraction.

At first visit, almost a quarter of patients were already under ARNI treatment, and 57.3% were receiving ACE-I or ARB.

At last follow-up, ARNI and SGLT2i use significantly increased to 60% and 64.5%, respectively (*p* < 0.01). Prescriptions for beta-blockers and mineralocorticoid receptor antagonists (MRA) were 85.5% and 55.5% at the initial visit, respectively, and did not show significant changes at the last visit. Patients with more than two drug classes increased from 53.6% to 68.2% (*p* < 0.05). Figure 1 shows changes in HF therapy between the first and last visit. Eleven patients were treated with cardiac resynchronization therapy (CRT) at the first visit, and five additional patients received this therapy during follow-up. Twelve patients carried an implantable cardiac defibrillator (ICD) at first visit, with no new implants during the time of study.

Multivariate analysis was performed to identify predictors of LVEF recovery. After adjustment, only age was associated with a lower probability of LVEF recovery, with no positive impact of the initiation of three or more HF medical therapies (Table 3).

### 3.3. Clinical Outcomes

Thirty-four patients (30.9%) experienced the composite primary outcome during follow-up (Table 4). The majority of these events (94.1%) consisted of the first episode of worsening heart failure. Principio del formularioFinal del formulario

Table 5 compares patients according to the occurrence of the primary outcome. Patients who did not experience the primary outcome were significantly more likely to receive treatment with SGLT2i (*p* < 0.001) and ARNI/ACE-I or ARB (*p* < 0.05) and were prescribed a greater number of disease-modifying HF medications (*p* < 0.05). These patients also exhibited a higher LVEF at the last follow-up visit (*p* < 0.05).

**Figure 2:**
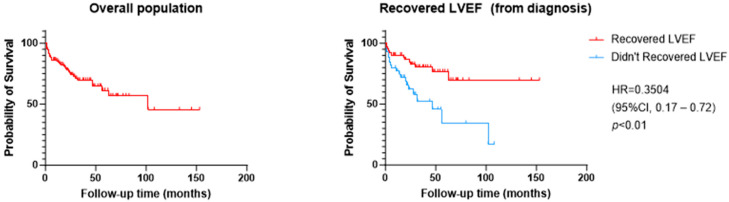
Kaplan–Meier curves for the composite primary outcome of time to cardiovascular death or the first episode of worsening heart failure, presented for the overall population and according to LVEF recovery. LVEF recovery was defined as LVEF increase of at least 10% from LVEF at diagnosis to a value over 50%.

**Figure 3:**
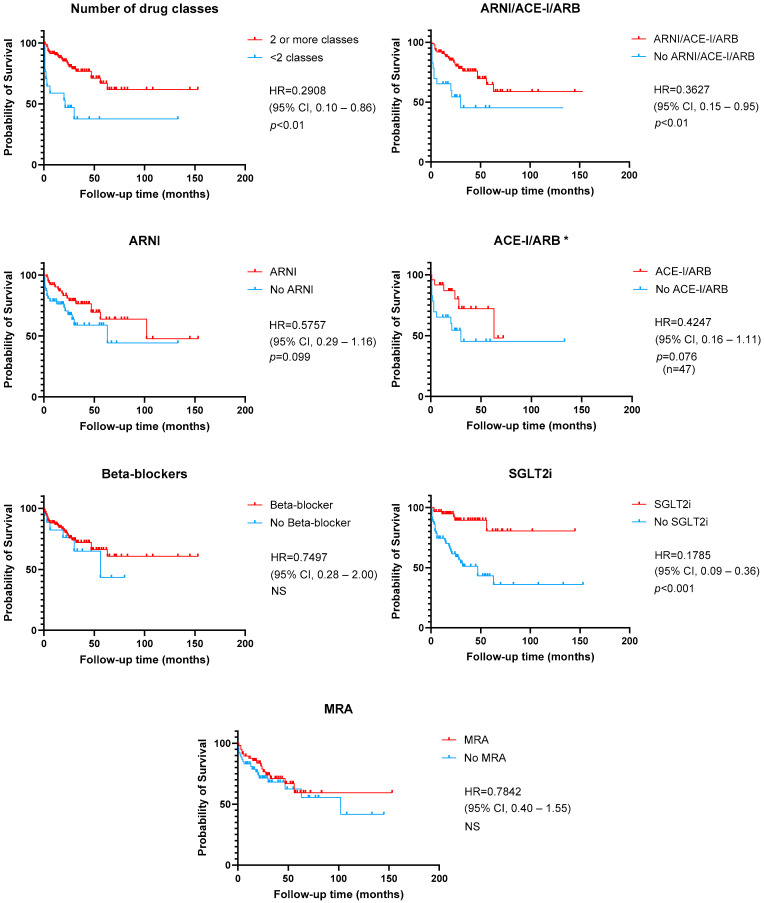
Kaplan–Meier curves for the composite primary outcome of time to cardiovascular death or the first episode of worsening heart failure, according to guideline-directed medical therapy. * Patients with ARNI were excluded. ACE-I—angiotensin-converting enzyme inhibitor; ARB—angiotensin-receptor blocker; ARNI—angiotensin receptor-neprilysin inhibitor; MRA—mineralocorticoid receptor antagonist; NS—non-significant; SGLT2i—sodium-glucose co-transporter 2 inhibitor.

Figure 2 and Figure 3 show a graphical representation of survival free from the primary outcome, depending on LVEF recovery, number, and type of HF disease-modifying drugs. Patients who recovered LVEF were at lower risk of the primary outcome (HR 0.35 [95% CI 0.17–0.72] *p* < 0.01). The use of two or more HF drug classes also had a positive effect on survival free from cardiac events (HR 0.29 [95% CI 0.10–0.86], *p* < 0.01). Regarding the implementation of GDMT, Kaplan–Meier curves and log-rank test also showed a benefit for vasodilator therapy (ARNI, ACE-I or ARB) (HR 0.36, *p* < 0.01) and SGLT2i use (HR 0.17 [95% CI 0.15–0.95], *p* < 0.001).

**Figure 4:**
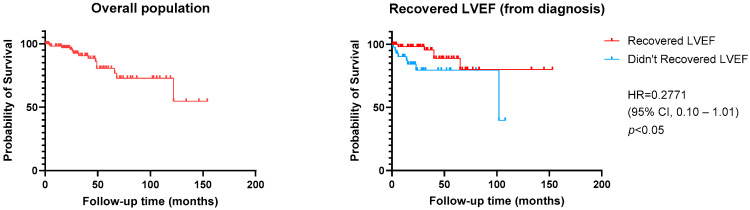
Kaplan–Meier curves for all-cause mortality, presented for the overall population and according to LVEF recovery. LVEF recovery was defined as LVEF increase of at least 10% from LVEF at diagnosis to a value over 50%.

**Figure 5:**
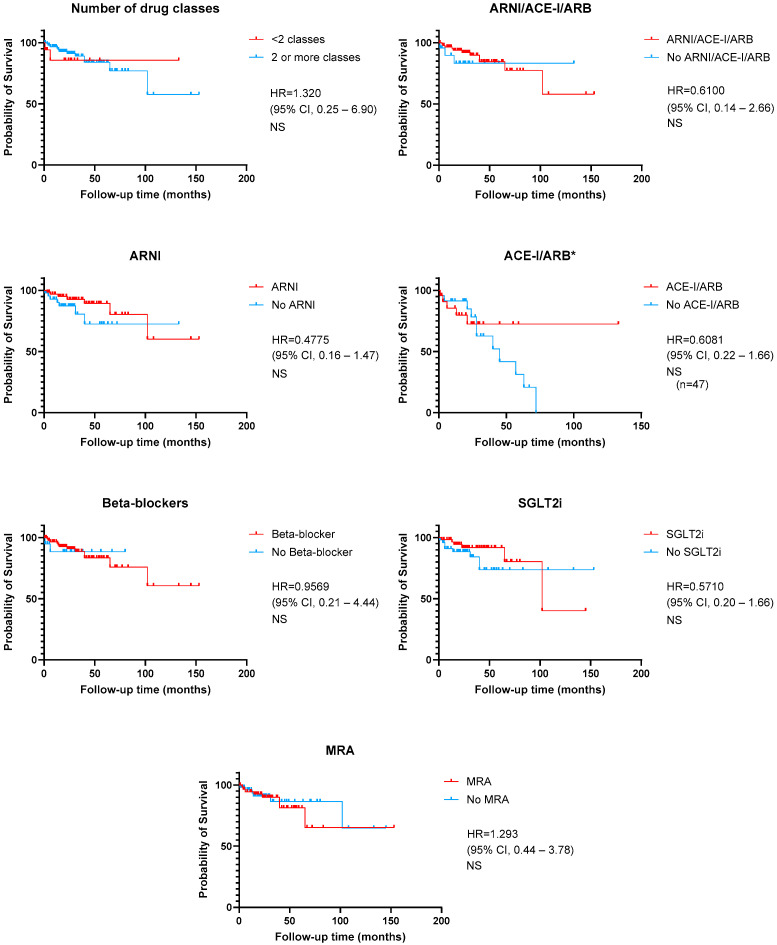
Kaplan–Meier curves for all-cause mortality, according to guideline-directed medical thearpy. * Patients with ARNI were excluded. ACE-I—angiotensin-converting enzyme inhibitor; ARB—angiotensin-receptor blocker; ARNI—angiotensin receptor-neprilysin inhibitor; GDMT: guideline-directed medical therapy; MRA—mineralocorticoid receptor antagonist; NS—non-significant; SGLT2i—sodium-glucose co-transporter 2 inhibitor.

Concerning overall mortality, survival analysis showed a benefit for patients who recovered LVEF (HR = 0.2771 [95% CI 0.10–1.01], *p* < 0.05) but no differences when analyzing each drug class (Figure 4 and Figure 5).

### 3.4. Treatment Changes After the First Episode of Worsening Heart Failure

**Figure 6:**
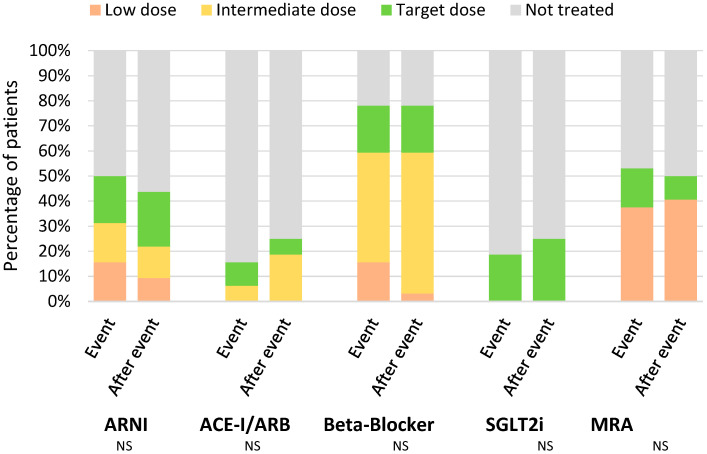
Changes in GDMT dosage after the first episode of worsening HF. ACE-I—angiotensin-converting enzyme inhibitor; ARB—angiotensin-receptor blocker; ARNI—angiotensin receptor-neprilysin inhibitor; LVEF—left ventricle ejection fraction; MRA—mineralocorticoid receptor antagonist; NS—non-significant; SGLT2i—sodium-glucose co-transporter 2 inhibitor.

No significant differences in HF treatment were observed after the first episode of worsening HF, with no optimization or dropout of drug classes one month after said event. Table 6 compares both time points, and Figure 6 shows the dosage for each individual medical therapy.

## 4. Discussion

This study reports the clinical and echocardiographic characteristics, treatment, and cardiac outcomes of a cohort of adults over 80 years with HFrEF. A high level of implementation of GDMT in older populations is feasible. LVEF recovery, a greater number of HF medical therapies, and treatment with a vasodilator agent or SGLT2i were associated with a reduction in the combined primary outcome of the first episode of worsening HF and cardiovascular death.

In this cohort, elderly patients with HFrEF who initiated a structured follow-up at the HF clinic received three or more HF drug classes in more than two-thirds of cases, with ARNI prescription in 60% and SLGT2i in 64.5% of patients at the last visit. The implementation of GDMT was higher than previously described in observational cohorts [14,15,16,17,18,19]. Real-world data usually show an underusage of HF therapies in the elderly in comparison to younger patients [20]. Also, recent registries such as CHECK-HF registry and EORP demonstrate an implementation of HF medical therapies significantly higher in patients aged less than 60 years and a decline in the use of GDMT with advanced age [14,16]. Available data about ARNI and SGLTi use in the elderly are very limited. In a recent prospective study of patients with a mean age of 83.3 years, only 14.3% of those with a theoretical indication for ARNI therapy were receiving the medication [18]. Similarly, in another recent registry of 364 octogenarians with HFrEF, only 15.1% were treated with SGLT2i and 26.4% with ARNIs [19].

Initiation and up-titration of HF medical therapies in older patients can be challenging. Elderly people with HF frequently present other major illnesses, with a high prevalence of chronic kidney disease, high cardiovascular risk, and frailty, which can interfere with drug efficacy and tolerability [5,21,22]. Comorbidities were frequent in our study, with more than half of the patients presenting chronic kidney disease. Indeed, chronic kidney disease is one of the main reasons for suboptimal use or non-up-titration of GDMT [23,24]. Also, polypharmacy increases the risk of adverse drug reactions and is associated with a higher treatment burden, which may be another potential explanation for the lower implementation of GDMT in the elderly [25,26]. Healthcare system characteristics and reimbursement policies of HF therapies can also have an impact on GDMT implementation [20]. The Spanish healthcare system usually covers the full price of prescription costs in the elderly, which may have favored GDMT prescription in our cohort. Follow-up at a dedicated HF clinic led by cardiologists may help explain the high rates of GDMT prescription in our cohort. First, older patients attending Cardiology HF clinics may be a selected, less frail group compared to the broader population of octogenarians. Second, cardiologists may be more attuned to the importance of GDMT implementation than geriatricians or general practitioners. Finally, dedicated HF clinics facilitate frequent consultations, offering a unique opportunity for careful up-titration of GDMT [13], which might not be possible in other settings. The application of our findings may, therefore, be limited outside this context; however, it does not invalidate the fact that GMDT is achievable with the proper resources. Also, our findings highlight the importance of referring older patients with HFrEF to dedicated HF clinics independently of their biological age if frailty is not a major concern.

Despite the complexities of GDMT implementation in older adults, its use was associated with a reduction in the primary outcome in our cohort. The implementation of two or more drug classes, or the prescription of any vasodilator agent (ACE-I/ARB/ARNI) or SGLT2i, was associated with a significant prognostic benefit. In line with our results, a growing body of evidence suggests that HF medical therapies are safe and benefit the elderly similarly to younger patients. Sacubitril/valsartan has proven to be superior to enalapril across the spectrum of age in a post hoc analysis of the PARADIGM-HF trial [9], and its use has been associated with a significant reduction in mortality in patients over 75 years [18]. Regarding ACE-I/ARB, post hoc analysis of major studies and meta-analysis has not found age-related heterogeneity [27,28,29], and no higher risk has been observed of syncope-related hospitalization when compared to younger patients [30]. SGLT2i constitutes the latest addition to the arsenal of HF therapies, so evidence concerning their use in the elderly is minimal. Prespecified subgroup analysis of major clinical trials did not show significant age-related heterogeneity [10,31]. Our study showed a notable protective effect of SLGT2i, which should be confirmed in further studies. The pleiotropic effects of this drug class may confer additional benefits in elderly patients by influencing other comorbidities such as reducing kidney disease progression [32] or atrial fibrillation risk [33]. In this cohort, beta-blockers or MRA administration was not associated with a risk reduction. Noteworthily, the prescription of both medications did not increase from the first to last visit in the HF clinic, although beta-blocker prescription was already high at first visit. It is well known that physicians have more concerns about beta-blockers and MRA adverse effects in the elderly population. The SENIORS trial, one of the few randomized clinical trials in an older population, showed that nebivolol was well tolerated and effective in reducing mortality and morbidity in patients of age >70 years with HF, regardless of the initial LVEF [34]. In this line, a subgroup analysis of the MERIT-HF trial described that metoprolol improved survival, reduced hospitalizations, and was safe and well tolerated in patients over 65 years [35]. An explanation for our different findings could be that patients in our study were older than those in the SENIORS trial (mean age 83 years versus 76 years). Indeed, some studies and meta-analyses have raised concerns about beta-blocker tolerability in very elderly patients, where difficulties in achieving target doses may diminish their impact [36]. In that sense, our cohort may not have been of large enough size to demonstrate an already attenuated effect. The small sample size of our cohort may also explain the absence of a positive impact of MRA administration. A meta-analysis of RALES, EMPHASIS-HF and TOPCAT did find a beneficial effect in mortality and morbidity in patients over 75 years [37]. However, studies display significant inter-study heterogeneity concerning mortality in patients with HFrEF over 75 years, suggesting that MRA’s effect may depend on the patient baseline characteristics [38]. Impaired renal function or the risk of hyperkaliemia may limit the use of MRA in frail, comorbid older patients. However, age alone should not be a barrier to prescribing MRA, as it often is currently [39]. Rather, a careful, individualized approach should guide ARM implementation. Our study suggests that there may be room for improvement concerning MRA prescription in the elderly, warranting close monitoring of renal function and potassium levels. Regarding concerns about worsening kidney function under GDMT, our study did observe a significant reduction in estimated GFR from the first to last visit, although it may be explained by age being included in the CKD-EPI creatinine equation [40].

Overall mortality analysis was limited by a relatively low mortality rate (fourteen deaths in more than two years of median follow-up), despite the mean age at first visit being very similar to the Spanish life expectancy [41]. As expected, half of the deaths were not secondary to cardiac causes [42]. This finding highlights the critical importance of comorbidities in this subset of patients and their associated competitive risks. Additionally, it underscores that the management of older patients with HFrEF and efforts to improve their quality of life should include strategies to address non-cardiac comorbidities.

Concerning implantable devices, CRT was not underused in our cohort when compared to the rates of CRT observed in recent major clinical trials including younger populations [10,43]. This strategy aligns with several retrospective studies, suggesting that older patients benefit similarly in terms of resynchronization response and reductions in HF-related hospitalizations [44,45,46,47]. In opposition, ICD implantation was infrequent in our cohort. ICD benefits are more controversial in the elderly, as the risk of arrhythmic death is lower with a higher competing risk of non-arrhythmic death, including non-cardiac deaths [48]. More studies are needed to assess ICD survival benefits in contemporary elderly people treated with GDMT.

During follow-up, most patients improved their functional class, LVEF, LVEDD, and degree of mitral regurgitation. A decrease in NT-proBNP levels was also observed, which is expected to be associated with a better prognosis [49,50]. It is noteworthy that only age demonstrated a negative impact on LVEF recovery, a finding consistent with previous reports [51]. This association may have attenuated GDMT’s effect on LVEF recovery in our cohort. However, this finding emphasizes GDMT’s effect on prognosis independently of LVEF recovery.

Recent guidelines suggest that a cardiac event, particularly a heart failure admission, is an opportunity for evidence-based treatment optimization [1]. Different than expected, no significant changes were observed when analyzing GDMT use before and after a first episode of worsening HF. Worse kidney function, hypotension, and higher frailty after admission could hinder this opportunity in the elderly.

Our study presented some limitations, mostly those inherent to a retrospective analysis, which only enables hypotheses generation. The small sample size may have prevented us from extracting further conclusions from data analysis. Also, lower-than-expected mortality in our cohort prevented a further analysis of the treatment effect on mortality. Frequent cardiology consultations may have impacted outcomes, such as worsening heart failure, which might have been more frequent in a less controlled environment. Also, follow-up in an HF clinic led by cardiologists may have introduced a selection bias towards more robust elderly patients, with frailer patients being followed at geriatric outpatient clinics. This may explain the high level of implementation of GDMT and low mortality rates. In this sense, data concerning frailty or comorbidity scores such as the Charlson comorbidity index could have provided valuable information. However, this finding does not undermine the fact that chronological age by itself should not be a limitation for GDMT use. In this sense, future studies should focus on the impact of frailty rather than age on GDMT’s effect. On the other hand, comparing with a younger matched control group could have strengthened our analysis by providing further insight into the specific effects of GDMT in the elderly. Future studies could use this approach to enhance our understanding of the matter. Ultimately, the relative importance of non-cardiac deaths highlights the differential features of this patient subset. Randomized clinical trials involving older patients are essential, with primary outcomes focusing on quality-of-life measures rather than overall mortality.

## 5. Conclusions

The optimization of HF treatment is achievable in older patients and may be associated with a reduction in cardiac events. Although further studies are needed, these results should encourage us to pursue a higher level of GDMT prescription in our seniors as it may benefit them significantly.

## Figures and Tables

**Table 1:** Sample description.

	n = 110
Female sex	34 (30.9%)
Age at Diagnosis (years) **	80.1 ± 6.1
Age at First Visit (years) *	82.9 ± 4.1
Duration of Follow-up (months) **	25.5 (14–47)
Hypertension	97 (88.2%)
Dyslipidemia	78 (70.9%)
Diabetes	46 (41.8%)
Smoking	
Yes	2 (1.8%)
Ex-smoker	45 (40.9%)
Moderate/severe COPD	5 (4.5%)
Sleep apnea with CPAP	9 (8.2%)
Chronic kidney disease (GFR < 45 mL/min/1.73 m^2^)	56 (50.9%)
LVEF at Diagnosis (%) *	32.4 ± 5.4
Etiology of heart failure	
Dilated cardiomyopathy, non-ischemic	41 (37.3%)
Ischemic	36 (32.7%)
Valvular	12 (10.9%)
Mix (valvular + ischemic)	13 (11.8%)
Amyloidosis	2 (1.8%)
Other	6 (5.5%)
Reason for referral to the HF clinic	
Heart failure admission	67 (60.9%)
Presence of symptoms or signs of HF	33 (30%)
Casual finding on echocardiogram	9 (8.2%)
Sudden cardiac death episode	1 (0.9%)

* Mean ± standard deviation; ** Median (percentiles 25–75) (minimum–maximum); CPAP—continuous positive airway pressure; COPD—chronic obstructive pulmonary disease; GFR—glomerular filtration rate; HF—heart failure; LVEF—left ventricle ejection fraction.

**Table 2:** Differences from first to last visit at the Heart Failure Clinic.

	First Visit	Last Visit	*p*
NYHA class			<0.01
I	19 (17.3%)	30 (27.3%)	
II	66 (60%)	66 (60%)	
III	25 (22.7%)	13 (11.8%)	
IV	0	1 (0.9%)	
ECG Rhythm			NS
Sinus rhythm	64 (58.2%)	59 (53.6%)	
Atrial fibrillation	44 (40%)	49 (44.5%)	
Auricular pacing	2 (1.8%)	2 (10.8%)	
QRS morphology			NS
LBBB	29 (26.4%)	32 (29.1%)	
RBBB	13 (11.8%)	17 (15.5%)	
IVCD	9 (8.2%)	4 (3.6%)	
Ventricular Pacing	14 (12.7%)	18 (16.4%)	
LVEF (%) ***	32.7 ± 8.4	45.2 ± 10	<0.001
LVEDD (cm) ***	5.6 ± 0.9	5.2 ± 0.9	<0.001
Grade III/IV mitral regurgitation	21 (19.1%)	6 (5.7%)	<0.001
Moderate/severe RV systolic disfunction	10 (9.1%)	12 (11.4%)	NS
NT-proBNP (ng/L) *	3091 (158–53,354)	1802 (145–19,509)	<0.001
Estimated GFR (mL/min/1.73 m^2^) ***	54.5 ± 18	47.2 ± 14.8	<0.001
ARNI	27 (24.5%)	66 (60%)	<0.001
ACE-I or ARB	63 (57.3%)	22 (20%)	<0.001
ARNI, ACE-I, or ARB	90 (81.8%)	88 (80%)	NS
Beta-blocker	94 (85.5%)	89 (80.9%)	NS
SGLT2i	26 (23.6%)	71 (64.5%)	<0.001
MRA	61 (55.5%)	59 (53.6%)	NS
Number of drug classes * 0/0 ** 1/1 ** 2/2 ** 3/3 ** 4/4 **	3 (0–4) (2–3) 3 (2.7%)/7 (6.4%) 13 (11.8%)/29 (26.4%) 35 (31.8%)/49(44.5%) 48 (43.6%)/19 (17.3%) 11 (10.0%)/6 (5.5%)	3 (0–4) (2–4) 4 (3.6%)/5 (4.5%) 11 (10%)/15 (13.6%) 20 (18.2%)/25 (22.7%) 44 (40%)/40 (36.4%) 31 (28.2%)/25 (22.7%)	<0.05/<0.001
ICD Primary prevention Secondary prevention	9 (8.2%) 3 (2.7%)	9 (8.2%) 3 (2.7%)	NS
CRT	11 (10%)	16 (14.6%)	NS

* Median (percentiles 25–75); ** With ARNI as neurohormonal antagonist; *** Mean ± standard deviation; ACE-I—angiotensin-converting enzyme inhibitor; ARB—angiotensin-receptor blocker; ARNI—angiotensin receptor-neprilysin inhibitor; CRT—cardiac resynchronization therapy; ECG—electrocardiogram; GFR—glomerular filtration rate; ICD—implantable cardioverter-defibrillator; IVCD—intraventricular conduction delay; LBBB—left bundle branch block; LVEDD—left ventricular end-diastolic diameter; LVEF—left ventricular ejection fraction; MRA—mineralocorticoid receptor antagonist; NS—non-significant; NYHA—New York Heart Association; RBBB—right bundle branch block; SGLT2i—sodium-glucose co-transporter 2 inhibitor.

**Table 3:** Predictors of LVEF recovery.

	Univariate		Multivariate	
	OR (CI 95%)	*p*	OR (CI 95%)	*p*
Age	0.82 (0.71–0.95)	0.009	0.82 (0.71–0.95)	0.010
Female sex	1.5 (0.61–3.69)	0.38		
Chronic kidney disease (GFR < 45 mL/min/1.73 m^2^)	0.79 (0.34–1.87)	0.60		
LVEF at diagnosis	1.00 (0.93–1.08)	0.90		
Ischemic cardiomyopathy	0.36 (0.12–1.04)	0.059	0.36 (0.12–1.07)	0.066
3 or more HF therapies	1.29 (0.50–3.31)	0.60		

GFR—glomerular filtration rate; HF—heart failure; LVEF—left ventricle ejection fraction.

**Table 4:** Primary outcome and mortality during follow-up.

Primary Outcome (Composite of First HF Hospitalization or CV Death)	34 (30.9%)
HF hospitalization/IV diuretics	32 (94.1%)
CV death	2 (5.9%)
All-cause death	14 (12.7%)
CV Death	6 (42.9%)
Cardiac death secondary to progression of heart failure	2 (14.3%)
Sudden/arrhythmic cardiac death	2 (14.3%)
Cardiac death (not arrhythmic or heart failure related)	2 (14.3%)
Non-cardiac death	7 (50%)
Death of unknown cause	1 (7.1%)

CV—cardiovascular, HF—heart failure, IV—intravenous.

**Table 5:** Differences in guideline-directed medical therapy and left ventricular ejection fraction according to the occurrence of the primary outcome.

	Primary Outcome		
	YES (n = 34; 30.1%)	NO (n = 76; 69.1%)	*p*
ARNI	17 (50%)	46 (60.5%)	NS
ACE-I or ARB	6 (17.6%)	18 (10.5%)	NS
ARNI, ACE-I, or ARB	23 (67.6%)	64 (84.2%)	<0.05
Beta-blocker	27 (79.4%)	64 (84.2%)	NS
SGLT2i	8 (23.5%)	51 (67.1%)	<0.001
MRA	16 (47.1%)	41 (53.9%)	NS
Number of drug classes *	2 (0–4) (1–3)	3 (1–4) (2–4)	<0.01
LVEF at first visit (%) **	33.1 ± 7.7	32.5 ± 8.8	NS
LVEF at last visit (%) **	41.2 ± 10.2	47.1 ± 9.4	<0.05

* Median (percentiles 25–75) (minimum–maximum); ** Mean ± standard deviation; ACE-I—angiotensin-converting enzyme inhibitor; ARB—angiotensin-receptor blocker; ARNI—angiotensin receptor-neprilysin inhibitor; LVEF—left ventricle ejection fraction; MRA—mineralocorticoid receptor antagonist; NS—non-significant; SGLT2i—sodium-glucose co-transporter 2 inhibitor.

**Table 6:** Changes in guideline-directed medical therapy after the first episode of worsening heart failure.

	Time of Event (n = 32)	One Month After (n = 32)	Change ** (%)	*p*
ARNI	16 (50%)	14 (43.8%)	−6.2	NS
ACE-I or ARB	5 (15.6%)	8 (25%)	+9.4	NS
ARNI, ACE-I, or ARB	21 (65.6%)	22 (68.8%)	+3.2	NS
Beta-blocker	25 (78.1%)	25 (78.1%)	0	NS
SGLT2i	6 (18.8%)	8 (25%)	+6.2	NS
MRA	16 (50%)	16 (50%)	0	NS
Number of drug classes *	2 (1–3)	2 (1–3)	0 ((−1)–2)	NS

* Median (percentiles 25–75); ** Changes over time ACE-I—angiotensin–converting enzyme inhibitor; ARB—angiotensin–receptor blocker; ARNI—angiotensin receptor-neprilysin inhibitor; MRA—mineralocorticoid receptor antagonist; NS—non-significant; SGLT2i—sodium–glucose co-transporter 2 inhibitor.

## Data Availability

The raw data supporting the conclusions of this article will be made available by the authors on request.
